# EpiContactTrace: an R-package for contact tracing during livestock disease outbreaks and for risk-based surveillance

**DOI:** 10.1186/1746-6148-10-71

**Published:** 2014-03-17

**Authors:** Maria Nöremark, Stefan Widgren

**Affiliations:** 1 Department of Disease Control and Epidemiology, SVA, National Veterinary Institute, 751 89 Uppsala, Sweden

**Keywords:** Cattle-transport, Control strategies, Decision support systems, Epidemics, Eradication programs, Network analysis, GIS

## Abstract

**Background:**

During outbreak of livestock diseases, contact tracing can be an important part of disease control. Animal movements can also be of relevance for risk-based surveillance and sampling, i.e. both when assessing consequences of introduction or likelihood of introduction. In many countries, animal movement data are collected with one of the major objectives to enable contact tracing. However, often an analytical step is needed to retrieve appropriate information for contact tracing or surveillance.

**Results:**

In this study, an open source tool was developed to structure livestock movement data to facilitate contact-tracing in real time during disease outbreaks and for input in risk-based surveillance and sampling. The tool, EpiContactTrace, was written in the R-language and uses the network parameters *in-degree*, o*ut-degree, ingoing contact chain and outgoing contact chain* (also called *infection chain*), which are relevant for forward and backward tracing respectively. The time-frames for backward and forward tracing can be specified independently and search can be done on one farm at a time or for all farms within the dataset. Different outputs are available; datasets with network measures, contacts visualised in a map and automatically generated reports for each farm either in HTML or PDF-format intended for the end-users, i.e. the veterinary authorities, regional disease control officers and field-veterinarians. EpiContactTrace is available as an R-package at the R-project website (http://cran.r-project.org/web/packages/EpiContactTrace/).

**Conclusions:**

We believe this tool can help in disease control since it rapidly can structure essential contact information from large datasets. The reproducible reports make this tool robust and independent of manual compilation of data. The open source makes it accessible and easily adaptable for different needs.

## Background

There are several reasons for preventing and controlling contagious diseases in livestock; securing food production, farmer economy, animal welfare and the zoonotic aspect. Both past and recent outbreaks have had large consequences both for the farming industry as well as other parts of the society [1,2]. Having tools ready to facilitate disease control and surveillance in critical stages of an outbreak can save time, aid in preventing further spread and thus minimise costs and consequences of the outbreak. Moreover, ongoing surveillance can contribute to early detection of disease outbreaks or assessing the disease status in a population. Applying a risk-based approach when sampling, i.e. searching in parts of the population where the likelihood of disease is higher or to identify strata where the consequences of disease introduction would be high, e.g. farms with many outgoing contacts can furthermore be a way to optimize surveillance resources [3,4].

Different diseases have different routes of spread. Yet, for most diseases, moving animals is considered to be one of the major risks for spreading disease between herds [5]. This is also one of the main reasons for registering transport of livestock in national databases, i.e. to enable contact tracing in case of an outbreak [6]. However, the data are not always structured in such a way that information relevant for contact tracing or design of surveillance programmes can be easily accessed by the end user.

**Figure 1:**
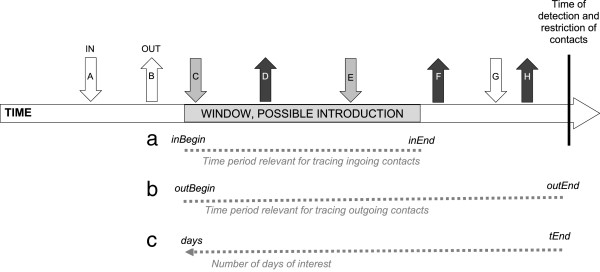
**Schematic illustration of the time window of possible introduction of a contagious disease to a farm, related to relevant time periods for contacts tracing.** The short arrows represent contacts; the light grey (C and E) represent ingoing contacts that could have introduced the disease; the dark grey (D, F and H) represent contacts which could have spread the disease. Contact A and G are ingoing contacts before and after the window of possible introduction and therefore not potential sources. Contact B is before the potential introduction and can therefore not have spread the disease. In relation to EpiContactTrace, the dotted lines represent how time periods can be indicated: **a)** the time period for ingoing contacts specified through dates for *inBegin* and *inEnd*, **b)** the time period for outgoing contacts specified through dates for *outBegin* and *outEnd*, **c)** the period can also be specified through a date *tEnd* and specifying the number of days of interest preceding that date, this will result in the same period for ingoing and outgoing contacts.

In the following text the word ‘farm’ will be used, meaning not only the premises but also the livestock present on the farm. Contagious diseases often spread from farm to farm in a sequential way and in contact tracing, both backwards and forward tracing is important, i.e. identifying farms from which infected animals may have come, and identifying farms which may have received infected animals. The time window of possible introduction of infection to the herd is relevant when determining contacts of interest. Animals introduced after the possible window of introduction can be excluded as the source, and animals leaving the herd before the possible introduction will not have spread the disease. Although, the window cannot always be determined, knowledge about the incubation period in combination with first appearance of symptoms can guide in the right direction. This is illustrated in Figure 1.

**Figure 2:**
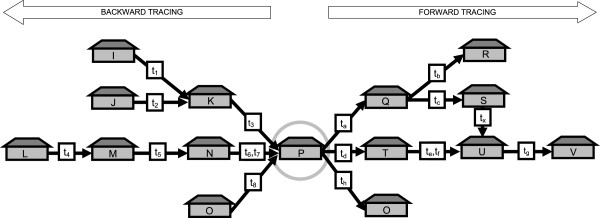
**A schematic illustration of backward- and forward contact-tracing, and the network measures *degree *and *contact chains*.** The encircled farm, P, represents the starting point for the contact tracing, in EpiContactTrace defined as the *root*. The arrows represent livestock movements, where *t* represents the point in time when the movement occurred. The left side shows ingoing contacts to P (backward tracing) and the right side outgoing contacts from P (forward tracing). The *in-degree*, i.e. direct ingoing contacts will be 3 (K, N and O) and correspondingly the *out-degree* will be 3 (Q, T and O). Since the same farm can be both among ingoing and outgoing contacts, this is exemplified with farm O. The measures *ingoing* and *outgoing contact chain* takes temporal aspect into account, i.e. the order in which the movements occurred. Given that t_1_ and t_2_ occurred before t_3_ and moreover that t_4_ occurred before t_5_ and that t_5_ occurred before t_6_ or t_7_, the *ingoing contact chain* will be 7. Given that t_a_ occurred before t_b_ and t_c_ and moreover that t_d_ occurred before t_e_ or t_f_, and that t_e_ or t_f_ occurred before t_g_, the *outgoing contact chain* will be 7. The movement arrow with time t_x_ illustrates the case where the same farm is included in different parts of the chain creating a cross-contact. Although appearing in different parts of the chain, a farm will only be counted once when indicating the measure *contact chain*.

The sequential spread of diseases through live animal contacts has been described by Webb and Dubé and co-workers, through the network measure *accessible world* and *infection chain*[7,8]. Correspondingly, the possible source farms have been described using the *ingoing infection chain*[9]. In this article, we hereafter refer to these measures as *outgoing contact chain* and *ingoing contact chain*, since they measure contacts and not confirmed spread of infection. These two network measures take the temporal aspect of movements into account and in combination with detailed information on the specific contacts identified, they are ideal for both backward and forward tracing of contacts through live animal movements during an outbreak (Figure 2). Moreover, the measures can be used to identify farms with many ingoing contacts or outgoing contacts, i.e. at high risk of introduction of disease or for spreading disease. In other words, information that could be relevant for risk-based surveillance and targeted sampling, or for targeted interventions during an outbreak. The information could also be of interest whenever animal movements are investigated as a risk factor for diseases occurrence. So far, many network articles published have been related to understanding structure of movements, modelling disease outbreaks, or to analyse movements post outbreak [10,11]. Although the effects of contact tracing on disease spread within a network has been investigated [12], there are fewer publications related to work providing applications for use during an ongoing outbreak [13]. However, the use of network measures for risk-based surveillance has been suggested by several authors [9,11,14,15] and also tested [16,17].

During outbreak contact tracing, one crucial source of information is structured interviews with farmers. Advantages with these types of interviews are that they can cover all relevant types of contacts for the disease in question, e.g. live animal, visitors or shared equipment. Disadvantages are that they are often time consuming and there is a need to get in touch with the farmer. Due to the sequential nature of contact tracing, failing to make contact with a farmer will delay the process of identifying other farms in need of tracing. Moreover, recall bias can affect the result. This is not necessary when using register data, if data are reported the contact information is not dependent on the farmer recalling the event. Moreover, tracing, even in several steps, can be done without having made contact with the farmer. However, when using register data, completeness and validity of data are important. For example temporal aspects, such as time from event to reporting, can affect the completeness of the data. Both structured interviews and register data are thus important sources of information during contact tracing. Unless there is perfect reporting, or perfect recall of all contacts by the farmer, one cannot replace the other and should instead be regarded as complementary to each other.

Tools for automatically generating reproducible reports have several advantages compared to first retrieving data and then manually including them in reports. Firstly there is a gain of time, secondly and most important, the reports always include the same content. This makes them less sensitive to change of personnel or human errors due to stress.

The aim with this project was to develop a tool that rapidly analyses, structures and visualizes animal movement data both for contact tracing during outbreaks and for risk based surveillance. Objectives were to produce reports for single farms, as well as datasets containing contact patterns for all farms in the dataset. Another objective was that the reports should be reproducible and user friendly for the end user, e.g. veterinary authorities, regional disease control officers and field-epidemiologist and veterinarians. The final objective was to make the tool accessible through open source.

## Implementation

The R environment [18] was used to develop a tool, EpiContactTrace (version 0.8.5), which performs network analysis, visualises and structures animal movement data (on individual or group level), and creates contact reports for use in outbreak contact tracing or risk-based sampling. EpiContactTrace can also be applied to other types of contact data, as long as the dataset contains information on source, destination and date. The package can be used from R, and most of the functionality is implemented in the R language. The package also makes extensive use of other R packages in order to add visualization features such as network plots [igraph0] [19] and spatial animation of contacts [animation, ggmap] [20,21]. Moreover, templates for generating reproducible contact tracing reports in PDF- or HTML-format use Sweave [22]. One critical issue during development was to make the implementation efficient for use on large datasets. Using the Rcpp package [23] the core network analysis code has been implemented in C++ [24] which significantly improves performance and speed.

### Network measures

The analytical basis in EpiContactTrace consists of the network measures *in-degree*, *out-degree, ingoing-* and *outgoing contact chains* (Figure 2) [7,9,25]. Analysis can be done for a single farm, a number of farms, alternatively for all farms present in the movement dataset. The contact network is analysed over a period of time defined by the user. Different time periods for ingoing- and outgoing contacts can be defined, and thus adapted to the window of possible disease introduction (Figure 1). Two different options are given; either specifying one date, *tEnd*_,_ and the number of days preceding this date, *days*. Alternatively, the starting and end- dates of the intervals are defined through *inBegin*, *inEnd* and *outBegin* and *outEnd*.

In infectious disease epidemiology, direct contact often means physical contact between two animals and indirect means contact via e.g. contaminated fomites. However, throughout the rest of this article direct contact means animal transport between two farms. Whereas indirect contact means sequential contact, e.g. farm A sending animals to farm B, farm B sending to farm C will result as an indirect contact from farm A to farm C. For the ingoing contacts, the search starts with the root farm, searching for all direct ingoing contacts during the relevant time period. This search identifies all source farms, i.e. all holdings that have a contact with the root farm as destination. The search is repeated for each of the extracted source farms and for their source farms, until there are no more sources within the time period. A modified depth-first approach is applied, i.e. since the temporal aspect is relevant for each part of the chain and since several contacts can have occurred between the same farms as well as cross-contacts in different parts of the chain (see example Figure 2), farms will be revisited, unless the relevant time period has already been examined in an earlier step of the process. This is in contrast to letting the system remember previously identified farms and not repeat the search, which could potentially lead to failure to identify existing contacts in the dataset.

Correspondingly, the outgoing contacts are identified, starting from the root and identifying all farms of destination.

### Output dataset and plots

The output of the analysis can be converted and thereafter exported in different ways; both a summary of the network measures and the complete network structure can be exported for further statistical processing. Alternatively, the package can generate a PDF- or HTML-report based on a specific farm, which can be useful for hands-on disease tracing in the field.

The output dataset called NetworkStructure, includes the structure of the network, with the following columns; root, inBegin, inEnd, outBegin, outEnd, direction, source, destination, distance. The distance measures the number of steps from the root, i.e. a direct contact has distance 1. The NetworkSummary summarizes for each root the four network measures; 1) *ingoing contact chain*, 2) *outgoing contact chain*, 3) *in-degree*, 4) *out-degree* for the given time period. Thus, the summary does not include the identities of the contacts. It is also possible to extract all contacts related to the specified roots (including all detail, i.e. individual identities, category, n, date of contacts), without information on the structure.

**Figure 3:**
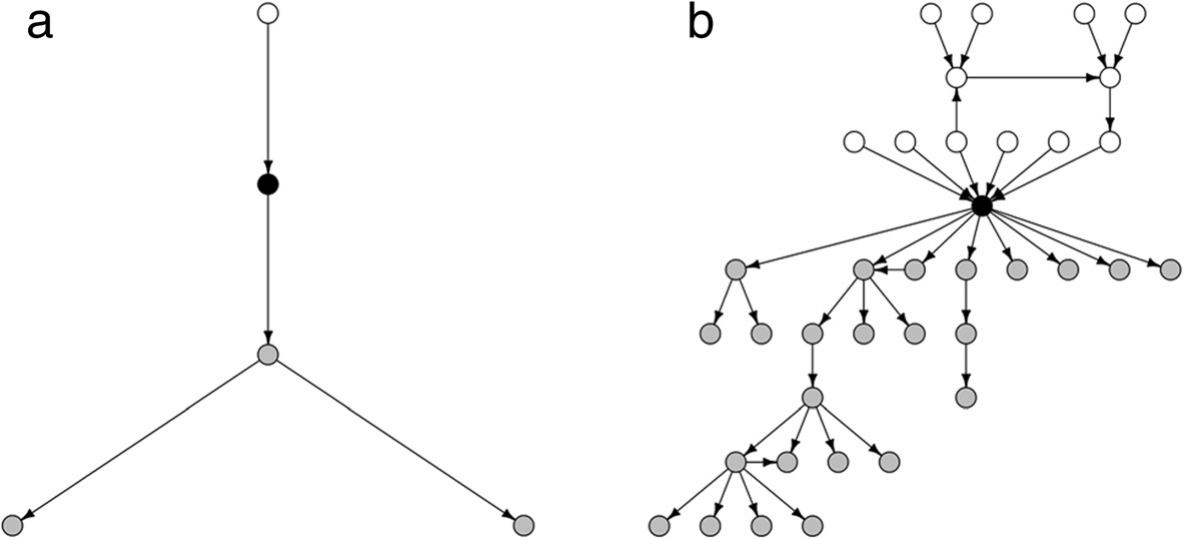
**Example plots of a simple (a) and a more complex (b) contact structure between farms.** The root is black, ingoing contact-farms are white, and farms reached through outgoing contacts are grey. Plots are generated using the EpiContactTrace example dataset *Transfers* with root 2838 and 2645.

Furthermore, a plot to visualize the contact structure can be created. A farm existing both as ingoing and outgoing contact will in the plot be represented both in the ingoing and the outgoing part of the plot. The primary purpose of this plot is to give an immediate visual impression of the size of the network, in other words, the purpose is not to identify individual nodes (Figure 3a and b). The root is black, nodes included in ingoing contacts are white and nodes included in outgoing contacts are grey. In the plot the contacts are structured at different levels, i.e. all nodes with direct contact are shown at the same horizontal level closes to the root; the ones with indirect contact one step away are shown on the next level and so on.

Moreover, whenever the geographical coordinates of the farms are available, the farms and the contacts can be plotted on a map to give insight of the spatiotemporal distribution of the contacts [animate, ggmap] [20,21]. Different time periods can be used for the plots, and plots can be shown in sequence like an animation. The plots can be useful in an outbreak situation to rapidly see which regions that have received animals from infected farms, or to get a general overview of animal movements between infected and non infected regions [26].

### Report

**Figure 4:**
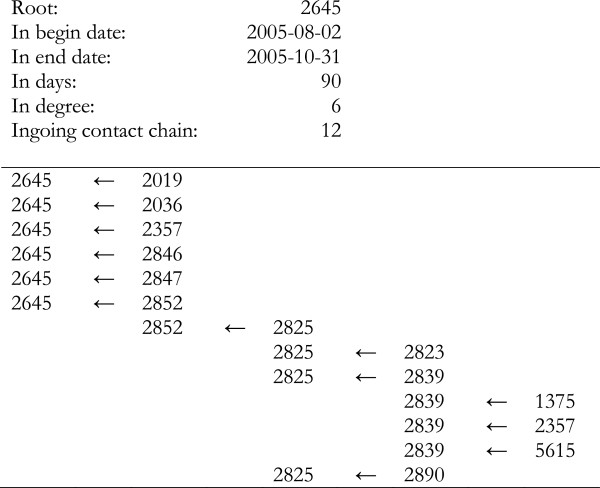
**Example showing the summary of the ingoing contact structure in the EpiContactTrace-report.** The example was generated using the EpiContactTrace example dataset *Transfers* with root 2645. The arrow describes the direction of the contact, i.e. the left hand side is the destination and the right hand side is the source. The interpretation of the summary is that 2645 have received animals from 5 farms (2019, 2036, 2357, 2846 and 2847) which have not received animals from other farms during the specified time period. 2645 has also received animals from 2852 which in turn has received animals from 2825, etc.

EpiContatTrace contains a report template [22] for the farm specific contact reports, this template can be adapted by the end user. However, in the default setting the report has the following layout; in the first part the contacts are visualised graphically in a plot (Figure 3a and b), as to give an immediate signal to the reader of the report of the number of ingoing and outgoing contact farms. In the following parts, the contact data are presented with different levels of detail split by ingoing and outgoing contacts. The first (Figure 4) includes collapsed data and the sequential contact structure at farm level (i.e. no information on individuals or dates). In this summary, the sequential structure of each part of the chain is included, and a farm that appears in several different parts of the chain can therefore be included more than once in the summary. The reason for this is to facilitate sequential tracing and getting an overview of each part of the chain. Using the example in Figure 2, the structure would be: i) P to Q, Q to R and S, S to U, and U to V, ii) P to T, T to U, and U to V. Consequently U and V will appear in two different parts of the chain since they could potentially have received infection through two different routes. After the summary all details of all contacts included in the *contact chains* are presented in text, i.e. date of contact and data on individual level when available.

As default setting the report is produced in HTML-format, which includes direct links from the summary to the detailed information. Alternatively a PDF-report is generated via TeX-format [27]. The report can be generated for one farm or for several farms simultaneously.

### Example

The following example shortly demonstrates how to use EpiContactTrace for contact-tracing of two specified farms. More details can be found in the package documentation which also contains other examples (e.g. how to specify different time periods for ingoing and outgoing contacts or how to get network measures for all farms within the dataset). The movement dataset used in this example, *transfers*, is contained in the EpiContactTrace-package. The dataset is fictitious data containing 70190 observations during the time period 2005-08-01 -- 2005-10-31 on the following 6 variables; source, destination, id, time, n, category (a definition of the variables is found below, see subsection Data).

The following two commands are used to load the EpiContactTrace package and the *transfers* dataset into R

The farm or farms of interest, here called root, are specified through an integer or character vector. This vector can consist of a single or several farm identifiers. For example, if the farms of interest are 2645 and 2838, this can be written as:

The time period is defined through specifying an end date and the length in days of the period of interest. The date can be specified in a Date format or as a character string in the format YYYY-MM-DD, for example for the last of October 2005, and the length of the period of ninety days,

The analysis of the two farms is executed through the following command

The following command produces a summary of network parameters *in-degree*, *out-degree*, *ingoing contact chain* and *outgoing contact chain*:

The contact tracing result can be viewed as a plot (see Figure 3a and b).

A report can be generated in either HTML or PDF file format, the reports are saved to the current working directory with the root as filename.

If only the network measures are of interest, these can be obtained most efficiently using the *NetworkSummary* directly. In this example, the network measures for all herds in the dataset over a period of 90 days prior to 2005-10-31 are calculated:

## Using of EpiContactTrace

### Prerequisites

#### Software

In order to use EpiContactTrace (version 0.8.5), R (2.15.1) must first be installed and then the R packages plyr (1.8) [28], R2HTML (2.2.1) [29], igraph0 (0.5.6) [19], animation (2.2) [20], ggmap (2.3) [21], Rcpp (0.9.13) [23] and EpiContactTrace (http://www.r-project.org/). Instructions for installing R and packages can be found in the online manual *R Installation and Administration*[30]. To be able to convert the LaTeX-file generated from the contact tracing report to a PDF-file, a TeX implementation must be installed on the computer. On Windows, MiKTeX can be used (http://miktex.org/).

#### Data

Farms must be identified either through an integer or character label. The movement data must contain; 1) source farm [integer or character], 2) farm of destination [integer or character], 3) the date of movement/contact [date format]. Furthermore, it is possible to include information on category [character] e.g. species of the animal, the number of animals in each movement [real] and identifiers for individual animals [character]. Data need to be structured with one movement/contact on each row. Data can be imported to the memory from different file-formats [31] however, import from a comma separated text file is the simplest way [32].

## Results

EpiContactTrace was tested during an FMD-outbreak contingency exercise in Sweden during 18-21st of October 2010. During this exercise a dataset with authentic cattle, pig, sheep and goat movements (during 90 days period) was obtained from the Swedish Board of Agriculture. An EpiContactTrace-report was generated for each farm for which there was a suspicion or confirmed case according to the predefined exercise scenario. Although not formally assessed, the involved veterinary officers found the reports informative and useful for their work. The experiences from the exercise were used in further development of the tool and report-template.

The first version of EpiContactTrace 0.6.8 was released on CRAN in June 2012. The 0.6.8 version did not use C++ for the network analysis, which has been implemented in the current version 0.8.5 (released on CRAN July 2013). The run-time performance for the NetworkSummary analysis has been compared between version 0.6.8 and version 0.8.5 on a Windows XP desktop computer (Intel® Core™ Duo CPU, 1.97 GHz, 3.25 GB RAM). The dataset *transfer* (including all herds) over 90 days ending at 2005-10-31 was used and the run-times were 1783.2 seconds (version 0.6.8) and 2.1 seconds (version 0.8.5), thus the NetworkSummary analysis on the current version is almost 850 times faster.

The package EpiContactTrace is open source licensed under the European Union Public Licence (EUPL) [33] and available at: http://cran.r-project.org/web/packages/EpiContactTrace.

## Discussion

To our knowledge, this is one of the first approaches to develop a tool for applying network analysis for livestock contact tracing in real time during ongoing outbreaks and producing reports for the end user, which can be either at central level or the veterinarian in the field [13]. Moreover, in an outbreak situation the tool can also be used for identify high risk farms with many direct or indirect contacts, both potential spreaders and receivers of disease. These farms may be relevant for targeted intervention, information campaigns or sampling during an outbreak. The tool specifically addresses the temporal and sequential aspects of animal movements which are relevant for disease spread. This is in contrast to static network measures, which do not take the temporal aspect into account [7,34].

Time can be a critical aspect during disease outbreaks, and during an outbreak the work load is often high both in the field and at central level, especially in the initial phase. Any tool that can facilitate contact tracing and help prioritise field resources in the work to control the disease can be beneficial. When designing the report template, the aim was to produce a user friendly report to avoid misunderstanding, with an immediate overview on the first page and then increasing level of detail to facilitate for the reader. An example is shown in Figure 3a and b, which illustrate two different farms where 3b has a more complex contact structure. Although the contacts in the example (Figure 2) were quite straightforward, this is not always the case; the contact structures can be complex, especially when the search covers a long period of time. For example, the same farm can be both among ingoing and outgoing contacts and this will often result in a quite chaotic plot. A design choice was therefore to separate nodes belonging to the ingoing and outgoing contacts in different parts of the plots, thus resulting in a farm possibly appearing both in the ingoing- and the outgoing part of the plot. Another part of complexity is when the same farm occurs several times in different parts of the *contact chains*. In this case, we chose to include the same farm several times in the summary. The reason for this choice was the sequential structure of spread and thus the sequential search when tracing disease. To clarify; investigation and sampling will often start with the direct contacts – if these are negative there will in most cases be no need to search further down the chain. Giving an example related to Figure 2; if farm T is negative there would be no need to sample farm U. However, farm U could potentially have been infected via farm Q and S, and therefore it is important not to dismiss farm U before all potential routes have been investigated. Consequently, farm U will appear more than once in the summary. In the last part of the report all details on all separate contacts are included. The reason for this is that the information on individual level can be of use when deciding which individuals to sample and when trying to further pinpoint exactly when disease was introduced.

The report-template can be adapted for different needs, e.g. the language of the headings can be changed, and regardless of the design the major advantages with automatically generated reports is that they can be produced quickly without first extracting data, and then manually compiling them in reports for field use. Moreover they are reproducible and thus always include the same content and are easy to recognize. This is also an advantage when working under time pressure.

Searching the contact structure of a single farm using EpiContactTrace is a rapid process; however, it requires access to data. Thus, ensuring that movement data can be accessed on short notice, and rapidly converted into the right format can be a useful part in outbreak preparedness. Another important aspect is having knowledge of existing bias in the raw data, such as missing reports, inconsistent reports or delay in reporting, and moreover being aware how these may affect the output of the analysis. The need for complementary interviews with farmers, hauliers etc. will vary depending on the amount of missing data and time from the movement occurred until data is available in the database.

As previously mentioned, many diseases can also spread through contacts other than animal movements, such as farm visitors, feed, vehicles or equipment. Other possible sources of information for contact tracing can be different types of registers, such as milk collection routes of dairy companies in addition to structured interviews. Whenever data on other types of relevant contacts are available (availability is likely to vary between countries) and there is knowledge about potential bias in the raw data, these can be added to the dataset and included in EpiContactTrace analysis. In other words, the potential use is not restricted to animal movement data.

The time-window of possible disease introduction is not always easy to identify and will differ depending on symptoms and incubation period. For example, a highly contagious disease with short incubation period and clear symptoms is not likely to remain unseen in the herd for a long time. For such a disease the possible window of introduction can be captured through starting with the time of appearance of symptoms and adding a relevant time period based on incubation (and a safeguard period if the very first case was not detected). This window will probably not be longer than a few weeks. Whereas for a disease with diffuse symptoms and long incubation period, such as scrapie or paratuberculosis, the window will be much more difficult to capture and contact tracing going years back in time can be relevant [35,36]. The tool takes this into account and the user can set the periods of search from days up to several years. Moreover, the window can either be specified by giving the starting and end date of the period, or alternatively with an end date and a number of days. For example, if the time period of interest for a given disease has been identified to 20 days before first appearance of symptoms, the user does not need to back-calculate which date this was but can just indicate the date of appearance of symptoms and 20 days. This reduces the risk for errors. Furthermore, since the last date of possible introduction will not always be the same as the last date for potential spread of infection, the time periods for ingoing and outgoing contacts can be specified independent of each other.

For use in disease surveillance, the tool enables identification of farms with many contacts – either directly through *degree* measures or sequentially through *contact chain*. This can be useful for risk-based surveillance when identifying parts of the population where the consequences, i.e. risk of spread would be large if infection would be present. Correspondingly, the tool can identify farms with many ingoing contacts and high likelihood of introduction. This can be useful for selection of strata to target with sampling, both in an emergency situation as mentioned above or in ongoing surveillance programs with the aim to increase chance of early detection or to estimate probability of freedom. Depending on the purpose of the surveillance, either only recent contacts or contact patterns for several years can be included. From previous studies of the Swedish cattle population it was clear that some farms with only one or few direct contacts had many indirect contacts [9], and basing decisions on sampling only on *degree* could therefore potentially miss risk farms. The measures *in-degree* and *ingoing contact chain* have been tested in a pilot study and although the diseases investigated also spread though other routes than live animals, there was an association between disease occurrence and number of direct and indirect sequential contacts [16]. The conclusion was that for diseases that spread through live animal contacts these measures can be useful in risk-based sampling [16].

The R environment was chosen since it is open source and integrates a suite of software for data manipulation and graphical display. The R environment also offers the possibility to share knowledge and add functionality through R packages [37] and also enables further development of code by others. Moreover, the environment offers a structure for building automatically generated reports [22].

There are many possibilities for further refinement of both the contact measures and the tool. One example could be to include measures containing the number of animals and the number of times contact has occurred, i.e. a differentiation between one animal moving at one occasion and 50 animals moving at ten occasions [38]. Another idea could be to add information on known risk factors or disease status. Furthermore, a user friendly web-application allowing direct use in the field could be beneficial. In summary we believe that EpiContactTrace can be of use both for contact tracing during outbreak and for risk-based surveillance and sampling and with the open source approach - we hope that extra functionality will suggested by others.

## Conclusions

We believe this tool can help in disease control since it rapidly can structure essential contact information from large datasets with livestock movement information. The reproducible reports make this tool robust and independent of manual compilation of data. The open source makes it accessible and easily adaptable for different needs.

## Availability and requirements

**Project name:** EpiContactTrace

**Project home page:**http://cran.r-project.org/web/packages/EpiContactTrace/ and https://github.com/stewid/EpiContactTrace

**Operating system(s):** Platform independent. The package works on all platforms supported by R.

**Programming language:** R

Other requirements (for EpiContactTrace version 0.8.5): R (2.15.1) and the following R packages; animation (2.2), igraph0 (0.5.6), plyr (1.8), R2HTML(2.2.1), ggmap (2.3), and Rcpp (0.9.13).

**License:** EUPL

Any restrictions to use by non-academics: no restrictions.

## Competing interests

The authors declare that they have no competing interests.

## Authors’ contributions

MN and SW contributed equally to the ideas and development of EpiContactTrace. SW did the programming. MN drafted the manuscript, SW critically revised the manuscript. Both authors read and approved the final manuscript.
